# Literature review of the epidemiology of influenza B disease in 15 countries in the Asia‐Pacific region

**DOI:** 10.1111/irv.12522

**Published:** 2018-03-07

**Authors:** Lance Jennings, Qiu Sue Huang, Ian Barr, Ping‐Ing Lee, Woo Joo Kim, Philippe Buchy, Melvin Sanicas, Bruce A. Mungall, Jing Chen

**Affiliations:** ^1^ Canterbury District Health Board Christchurch New Zealand; ^2^ WHO National Influenza Centre Institute of Environmental Science and Research Porirua New Zealand; ^3^ WHO Collaborating Centre for Reference and Research on Influenza Melbourne VIC Australia; ^4^ Department of Pediatrics National Taiwan University Children's Hospital Taipei Taiwan; ^5^ Department of Internal Medicine Korea University Guro Hospital Seoul Korea; ^6^ GSK Singapore Singapore; ^7^ Present address: Sanofi Pasteur Asia & JPAC Region Singapore Singapore

**Keywords:** Asia‐Pacific, epidemiology, influenza B, literature review, seasonality, vaccine mismatch

## Abstract

Influenza control strategies focus on the use of trivalent influenza vaccines containing two influenza A virus subtypes and one of the two circulating influenza type B lineages (Yamagata or Victoria). Mismatches between the vaccine B lineage and the circulating lineage have been regularly documented in many countries, including those in the Asia‐Pacific region. We conducted a literature review with the aim of understanding the relative circulation of influenza B viruses in Asia‐Pacific countries. PubMed and Western Pacific Region Index Medicus were searched for relevant articles on influenza type B published since 1990 in English language for 15 Asia‐Pacific countries. Gray literature was also accessed. From 4834 articles identified, 121 full‐text articles were analyzed. Influenza was reported as an important cause of morbidity in the Asia‐Pacific region, affecting all age groups. In all 15 countries, influenza B was identified and associated with between 0% and 92% of laboratory‐confirmed influenza cases in any one season/year. Influenza type B appeared to cause more illness in children aged between 1 and 10 years than in other age groups. Epidemiological data for the two circulating influenza type B lineages remain limited in several countries in the Asia‐Pacific, although the co‐circulation of both lineages was seen in countries where strain surveillance data were available. Mismatches between circulating B lineages and vaccine strains were observed in all countries with available data. The data suggest that a shift from trivalent to quadrivalent seasonal influenza vaccines could provide additional benefits by providing broader protection.

**WHAT THIS PAPER ADDS**

- By bringing together data from across the region, we show that influenza contributes to the public health burden in Asia‐Pacific countries, with a variable, but substantial proportion due to influenza B. Influenza vaccination policies are needed in Asia‐Pacific countries, and the use of quadrivalent influenza vaccines could provide additional benefits.

## 1 INTRODUCTION

Epidemic influenza causes global public health burden each season. The World Health Organization (WHO) estimates that influenza severely affects between three and five million individuals each year and causes between 250 000 and 0.5 million deaths.^1^ The influenza attack rate is highest in children, while complications including hospitalization and death occur most frequently in elderly individuals.^1^ Other specific high‐risk groups prioritized by WHO for vaccination include pregnant women, the highest priority group, followed by individuals with a compromised immune system and individuals with comorbidities such as pulmonary or cardiac disease.^2^

Influenza type A and B viruses cause the vast majority of influenza disease in humans, and infection is preventable by vaccination. The relative proportion of influenza cases caused by type A and type B strains varies annually, reflecting antigenic drifts in the predominant strains and the host's level of immunity. In the last decade, influenza A viruses represented by three subtypes (A/H3N2, A/H1N1, and A/H1N1pdm09) have predominated during influenza seasons. Influenza B viruses are represented by two separate lineages (B/Victoria and B/Yamagata) that co‐circulate. In the 1980s, the B/Yamagata/16/88 lineage and its variants spread worldwide, whereas B/Victoria/2/87 lineage viruses remained geographically restricted to Asia during the 1990s for reasons not wholly understood. In 2002, the B/Victoria lineage strains spread to the rest of the world.^3^, ^4^, ^5^, ^6^ Influenza B has been isolated in up to 44% of laboratory samples in the United States from 2001 to 2002 through 2010‐2011 seasons (excluding the 2009‐2010 pandemic period), and in up to 60% of samples in Europe during the same period, with a seasonal average of 24% and 23% of samples, respectively.^7^

Seasonal influenza vaccines are modified annually to include those antigenic variants that are likely to predominate in the following influenza season. Vaccine strain selection is performed by the WHO using data from the Global Influenza Surveillance and Response System, a network of over 140 institutions in 111 countries.^8^ The B/Yamagata and B/Victoria influenza strains are antigenically distinct, and vaccines using one lineage induce only low levels of cross‐protection to the other lineage.^9^, ^10^ Trivalent seasonal influenza vaccines only contain one influenza B lineage, and it is not always possible to predict which B lineage will predominate during the next influenza season.^11^ Mismatch between the vaccine lineage and circulating influenza B lineage has occurred regularly, which can have a significant impact on influenza vaccine efficacy.^4^, ^12^, ^13^, ^14^ Since 2012, the WHO has recommended the inclusion of strains from both B lineages in quadrivalent seasonal influenza vaccines.^15^

Co‐circulation of both influenza type B lineages has also been documented throughout South‐East Asia and Oceania.^5^ The use of influenza vaccine in many Asia‐Pacific countries is limited, and the potential impact of quadrivalent influenza vaccines on illness and hospitalization rates in these countries is not known, but is also likely to be low.^16^ To obtain an epidemiological view of influenza type B in the Asia‐Pacific region, we conducted a review of the available literature. We attempted to identify periods of influenza B lineage mismatch between vaccine and circulating strains in 15 countries within the Asia‐Pacific region to better inform health authorities of the potential benefits of quadrivalent influenza vaccines for protection against seasonal influenza.

## 2 METHODS

**Figure 1:**
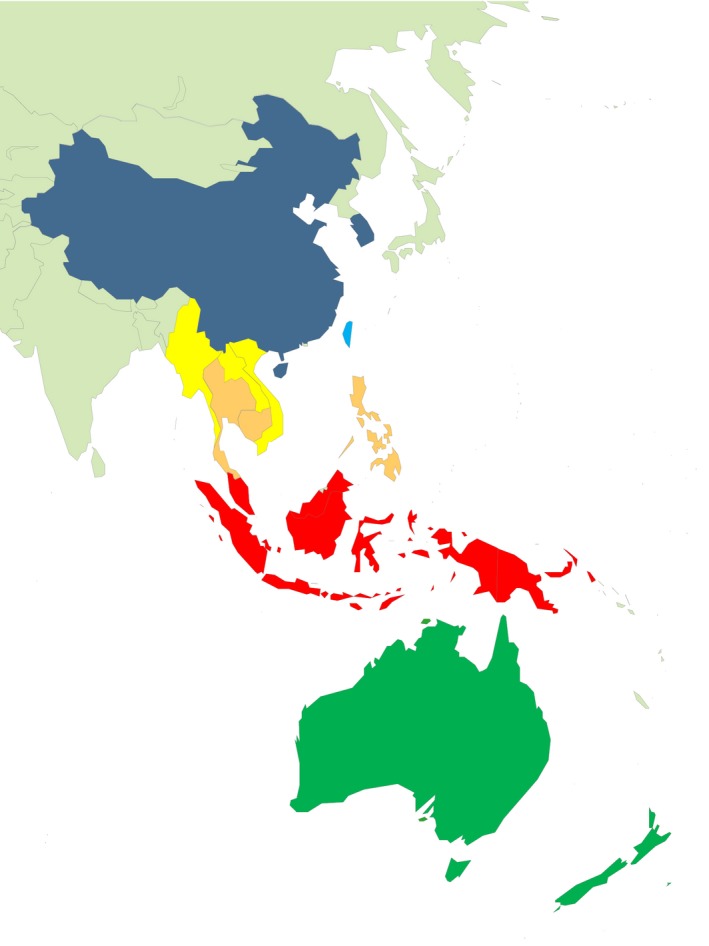
Countries included in the review according to climate. Northern Hemisphere: temperate = dark blue, subtropical = light blue, subtropical to tropical = yellow, tropical = orange. Southern Hemisphere: tropical = red, temperate = green

Fifteen countries were selected from within the Asia‐Pacific region traversing all climatic zones (Figure 1). These included Northern Hemisphere countries: China (temperate to subtropical climate), South Korea (temperate climate), Taiwan (subtropical climate), Laos, Myanmar and Vietnam (subtropical to tropical), Cambodia, Thailand, and the Philippines (tropical); countries in the Equatorial region: Indonesia, Malaysia, Singapore, and Papua New Guinea (tropical); and countries in the Southern Hemisphere: Australia and New Zealand (temperate).

### 2.1 Search strategy and selection criteria

Articles were identified from PubMed and the Western Pacific Region Index Medicus (WPRIM) using search strings comprised of terms that identified influenza, the selected countries of interest in the Asia‐Pacific region, and the epidemiology/burden of disease (Appendix S1). The search covered publication dates between January 1990 until 11 April 2016 and was limited to articles published in English language. Titles and abstracts were screened for relevance: That is, reported outcomes related to seasonal influenza in humans and in a country of interest. Articles were excluded if they reported pharmacokinetic or pharmacodynamic studies, case reports, case series, clinical trials, or meta‐analyses. Articles were also excluded if they reported features of influenza pathophysiology, treatment, or diagnosis, or if they reported data in fewer than 30 patients. Publications without abstracts were only reviewed if the title fitted the review objectives.

Full‐text articles were reviewed to assess their relevance and methodological quality. Articles were excluded if the method sections were insufficiently described; if the content did not provide relevant information to the review objectives; if the article reported “pneumonia and influenza” as a combined outcome (unless pneumonia was described as a complication of influenza), outcomes from mathematical models; and if no quantitative data could be retrieved. Gray literature including WHO websites, local ministries of health, and WHO vaccine recommendations was also assessed for relevance. Extracted data included information on epidemiology and circulating strains. We did not collect clinical criteria or clinical case definitions of influenza, influenza‐like illness (ILI), febrile illness, acute respiratory infection (ARI) disease, or severe acute respiratory illness (SARI) used in individual studies. Nor did we specify the methods for selecting cases for specimen collection or the influenza‐testing method used for laboratory diagnosis. In our review, “laboratory‐confirmed influenza” or a “positive sample” refers to a case of influenza confirmed by the method stated in the reporting paper.

An influenza B mismatch was defined as the circulating influenza B virus lineage strains differing from the B lineage representative strain included in the WHO‐recommended influenza vaccine composition for that season. When <20% of the circulating influenza B strains differed from the WHO‐recommended vaccine strain, we arbitrarily considered the degree of mismatch to be “low.” A difference between 20% and 40% was considered as partial mismatch. We considered a significant mismatch as >40% and complete mismatch when ≥95% of the circulating influenza B lineage strains did not belong to the trivalent vaccine lineage.

The initial literature search in 2013 was conducted by Pallas Health Research and Consultancy B.V., the Netherlands. Quality control activities included review of the first 30% of titles and abstracts and of the first 10% of full‐text articles in duplicate by two independent researchers from Pallas. Any disagreements were adjudicated by a third researcher. The search was updated in 2016 by BM, and the articles were selected by BM and JC.

Ethics approval was not required for this study. The majority of relevant publications concerned surveillance or other observational epidemiological studies for which no standard quality checklists are currently available.

## 3 RESULTS

**Figure 2:**
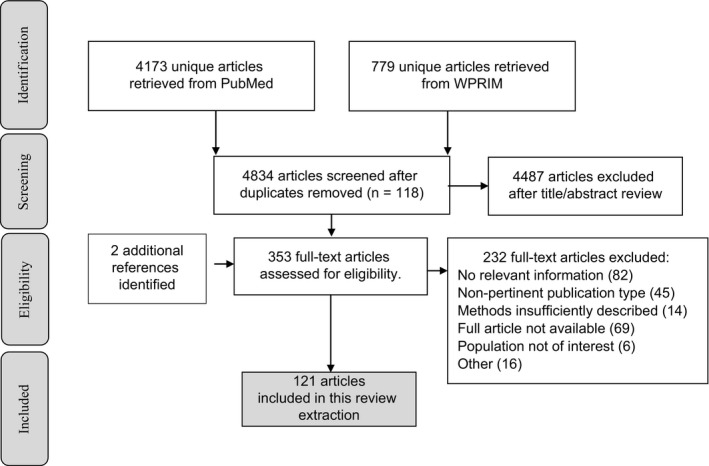
Article selection procedure. WPRIM: Western Pacific Region Index Medicus. Other includes studies with sample size <30, same data (or better quality data) provided in other articles, article in Korean language

There were 121 English language articles included in the review (Figure 2), of which 120 articles provided information on influenza B strains as a proportion of all laboratory‐confirmed influenza from data collected between 1990 and 2015 (Table 1). Most assessed specific, but diverse, populations of interest, such as patients hospitalized for respiratory tract infections (RTI), patients in intensive care, and respiratory samples from in/outpatients with a broad range of underlying respiratory syndromes. In many studies, the age range of subjects/samples was not specified. Many studies included patients with diagnoses of low specificity for influenza such as “febrile illness,” “ILI,” and “acute lower respiratory tract infection” (ALRTI). There were 102 prospective studies (two studies included both prospective and retrospective components). Sample sizes in individual study groups ranged from 26 to more than 300 000.

**Table 1:** Influenza surveillance systems and estimates of influenza vaccine coverage in fifteen Asia‐Pacific countries

Country	Influenza surveillance systems	Doses distributed per 1000 population^a^	Influenza vaccine coverage	Recommendation	National immunization program	Recommending body
Australia^19^, ^95^, ^96^	State and Territory‐based Influenza surveillance: General Practice Sentinel Surveillance, Influenza Complications Alert network, Emergency Department, Hospital Mortality, and Laboratory surveillance	299.73 (2013)	39% of adults ≥ 18 years, 73% of 65+ (2014)	All individuals from 6 months	Reimbursed for 65+, Aboriginal and Torres Strait Islander people, pregnant women, chronic disease	Australian Technical Advisory Group on Immunization
Cambodia^32^, ^33^, ^97^	National Influenza Center: ILI sentinel surveillance and hospital‐based ALRI surveillance since 2006	2.21 (2013)	12% among ILI patients (May 2010‐Dec 2012)	None	No	Ministry of Health
China^17^, ^18^, ^19^, ^98^, ^99^	National Influenza Center: ILI sentinel and hospital‐based surveillance since 2000	7.81 (2013)	9.0% (2011) overall, 7.4% in 60+years 9.4% chronic disease 9.5% healthcare workers 26% in children 16 doses/1000 in Shanghai (Zhao 2015) Highest (108 doses/1000 population) in Beijing where vaccination is free or subsidized for some age groups	Healthcare workers, elderly 60+, pregnant women, children from 6 years	No national recommendation	National Health and Family Planning Commission
Indonesia^45^, ^100^	ILI sentinel surveillance, enhanced surveillance for seasonal, and avian influenza in East Jakarta	1.81 (2011)	No reports identified	Prior to Hajj	No	Indonesian Technical Advisory Group on Immunization, Indonesian Pediatrician Association, Adult Immunization Task Force, Medicine Specialist Association of Indonesia
Laos^19^, ^98^	Laboratory‐based surveillance from 2007, hospital‐based surveillance (3‐8 sites) of pneumonia from 2008. Department of Health	15.21 (2013)	No reports identified	Healthcare workers, elderly 50+, pregnant women, chronic disease, children	?	Ministry of Health
Malaysia^19^, ^98^, ^101^	Sentinel surveillance for ILI (OPD) and SARI (hospital) Coordinated by the Surveillance Sector, Disease Control Division, Ministry of Health	7.48 (2013)	7.2% of HCW in 2011	Healthcare workers, chronic disease, elderly with ≥ 1 chronic disease, Hajj pilgrims	No	Ministry of Health
Myanmar^100^	None	None recorded (2001)	No reports identified	‐	No	‐
New Zealand^19^, ^102^, ^103^	National Influenza surveillance: General Practice Sentinel Surveillance of ILI, Laboratory‐based surveillance	312.5 (2013)	67.5% of 65+ (2014), 66% of healthcare workers (2015)	All individuals from 6 months	Reimbursed for Children 6 months‐ < 5 with respiratory condition, 65+, pregnant women, chronic disease	Ministry of Health
Philippines^19^, ^98^, ^104^	Sentinel surveillance for ILI (outpatient) and SARI (outpatient and hospital‐based), laboratory‐based surveillance, Department of Health	35.28 (2013)	No reports identified	Healthcare workers, chronic disease, elderly 60+, pregnant women, children (6 m ‐ 18 years)	Reimbursed for elderly 60+ indigent population in 2014‐15	Department of Health. Input and advice from Philippine Society for Microbiology and Infectious Diseases, Pediatric Infectious Disease Society of the Philippines, Philippine Foundation for Vaccination
PNG^19^, ^98^	PNG National Influenza Centre, ILI surveillance in 2 hospitals	0 (2013)	No reports identified	None	No	Ministry of Health
Singapore^19,62,98,105,106^	Outpatient (polyclinic and ED) attendance and admissions for ARI, influenza virus surveillance by National Influenza Centre	78.48 (2013)	30.6% among diabetics (2007)	Healthcare workers, chronic disease, elderly 65+, pregnant women, children (6 m ‐ 5 years), Children 6‐18 m on long‐term aspirin, residents of nursing homes	No	Ministry of Health
South Korea^19^, ^107^, ^108^, ^109^, ^110^, ^111^	Korean Influenza Surveillance System (KISS) (ILI surveillance at 630 sentinel clinics and 396 laboratories), Hospital‐based Influenza Morbidity and Mortality (HIMM) and inpatient surveillance (ED surveillance)	335.7 (2013)	34.3% in the general population, 61.3% high‐risk groups (2005), 82.4% of 65+ year‐olds (2016)	Elderly 65+, pregnant women, chronic diseases, children 6 m‐5 years, residents of nursing homes and long‐term care facilities, healthcare workers, children 6 m‐18 years on long‐term aspirin, adults 50‐64 years, household contacts and caregivers those at risk	Reimbursed for 65+, financially vulnerable, handicapped individuals, soldier, children 6‐59 ms	Centers for Disease Control and Prevention
Taiwan^112^, ^113^	Taiwan National Influenza Surveillance System (NISS): laboratory surveillance, sentinel ED and OPD ILI surveillance, case‐based surveillance of influenza with complications and deaths		40% <3 years of age 80% in adults (2012)	All individuals from 6 months	Reimbursed for high‐risk groups and 65+ years	Centers for Disease Control
Thailand^72^, ^100^, ^114^, ^115^, ^116^	Sentinel surveillance for ILI (outpatient) and SARI (hospital‐based), Thai National Institute of Health	103.3 (2011)	30% of 6 m‐2 years (2011‐2012), 9% of ≥50s hospitalized with ARI	Healthcare workers, 65+ underlying disease, children 6 m‐2 years, pregnant women, obese	Reimbursed for high‐risk groups and 65+ years	Ministry of Public Health
Vietnam^59^, ^98^, ^100^	National Influenza Surveillance System (NISS) since 2006. Sentinel surveillance for ILI (outpatient)	None recorded	No reports identified	Healthcare workers, chronic disease, elderly 65+, children from 6 m	No	Ministry of Health

CAP, community‐acquired pneumonia; ED, emergency department; HCAP, healthcare‐associated pneumonia; ICU, intensive care unit; ILI, influenza‐like illness; LRTI, lower respiratory tract infections; NA, aot applicable; OPD, outpatient department; PNG, Papua New Guinea; P&I, pneumonia and influenza; RTI, respiratory tract infection; SARI, severe acute respiratory illness.

a Pertains to trivalent influenza vaccines.

### 3.1 Influenza surveillance and vaccine coverage in Asia‐Pacific countries

With the exception of Myanmar, all of the countries studied use a sentinel site approach for influenza surveillance (Table 1). All countries with a surveillance program in place monitor ILI cases (ARI in Singapore and pneumonia in Laos instead of ILI) presenting to general practices or hospitals. In Myanmar, epidemiological studies funded by grant programs in Japan were conducted in two hospitals and general practice clinics in Yangon.

Six countries (Australia, New Zealand, South Korea, Taiwan, Thailand and the Philippines) have/have had a publicly funded national seasonal influenza immunization program, and in all cases, this is/was limited to at‐risk groups (Table 1).

China, Indonesia, Laos, the Philippines, Singapore and Vietnam provide recommendations for immunization of risk groups (and Malaysia recommends vaccination of Hajj pilgrims) outside the national schedule. No policy for influenza vaccine use exists for Papua New Guinea, Cambodia, or Myanmar.

In countries with a policy to provide free vaccine for at‐risk groups, vaccine uptake among these groups has been substantial: Approximately 73% of 65+‐year‐olds in Australia, 67.5% in New Zealand, 80% of adults in Taiwan, and 82.5% in 65+‐year‐olds in South Korea were reported to have received influenza vaccine (Table 1). In China, influenza vaccine coverage was reported as 26% in children and 7.4% in 60+‐year‐olds, but regional differences may exist due to subsidization of influenza vaccines in some regions.^17^, ^18^ Approximately 300 doses of influenza vaccine were distributed per 1000 population in Australia and New Zealand, while fewer than 10 doses per 1000 population were distributed in Cambodia, China, Indonesia, Malaysia, and Papua New Guinea, suggesting that influenza vaccine use is negligible in these countries.^19^

### 3.2 Epidemiology of influenza type B in Asia‐Pacific countries

#### 3.2.1 Australia

Laboratory surveillance conducted by the WHO collaborating center and National Influenza Centers showed that during the period from 2005 to 2015, influenza B viruses predominated in two years: in 2005, 67% of circulating influenza viruses were type B, while 51% of the influenza B viruses mismatched the B strain contained in the seasonal influenza vaccine; in 2015, 62% of circulating influenza viruses were type B with a partial (36%) mismatch to the trivalent vaccine influenza B component (Table 3).^20^, ^21^, ^22^

We identified 18 articles that described influenza B in Australia reporting studies conducted from 1991 until 2015. Most of the articles reported data from laboratory‐based influenza surveillance or clinic/hospital‐based surveillance in various states/territories. Some of the studies were conducted in the same/overlapped seasons but in different groups of patients.

One study in 2002‐2003 found that the proportion of influenza B was higher among influenza‐positive specimens from state‐wise influenza surveillance program of ILI patients at sentinel general practitioner (GP) clinics than among influenza‐positive patients hospitalized with respiratory illness (11.5% vs 1.8%).^23^ Two studies conducted in 2006 reported higher influenza detection rate among outpatients with ILI comparing to hospitalized children with ILI, while the proportion of influenza B was higher among laboratory‐confirmed influenza inpatients comparing to outpatients.^24^, ^25^

Two studies with overlapping study period in 2007 found significantly higher influenza‐positivity rate among ILI patients presenting to GP clinics than children with ARI presenting to hospital (46.9% vs 12.2%). The difference between the proportion of influenza B among the two groups of patients in these two studies was much smaller (12.7% vs 18.9%).^26^, ^27^ Comparing to these two studies, another study encompassing the same study period reported a much lower influenza B proportion among hospitalized children with laboratory‐confirmed influenza (2.5%).^28^

In 2012, GP clinic‐based surveillance in Western Australia and Victoria reported similar influenza detection rate among ILI patients.^29^, ^30^ The proportion of influenza B among influenza‐positive samples obtained from ILI patients was significantly higher in Western Australia than in Victoria (43% vs 13.6%).

#### 3.2.2 Cambodia

Since 2006, the National Influenza Centre in Cambodia carried out sentinel site‐based ILI surveillance and hospital‐based ALRI surveillance. Influenza viruses were detected from 5.8% to 18.7% of ILI patients between 2006 and 2011, and 1.4% to 3.6% of ALRI patients from 2007‐2010. Proportion of influenza B among all influenza‐positive samples ranged from 12.6% in 2009 to 64.8% in 2011. Influenza type A and B circulated year‐round in Cambodia with peak activities during the rainy season between June and November.^31^, ^32^, ^33^

#### 3.2.3 China

National surveillance of ILI has been carried out by the Chinese CDC in 95 sentinel hospitals in Northern provinces and 99 hospitals in southern provinces since 2000. From 2005‐11, 64 306 laboratory‐confirmed influenza cases were recorded by the ILI surveillance in the North. In Southern China, 122 215 laboratory‐confirmed influenza cases were confirmed by the ILI surveillance during 2006‐12. Around 30% of all positive samples were influenza B.^34^

Twenty‐three articles reported on influenza B in China from 1995 until 2014 (Table 2). Most of the articles reported clinic/hospital‐based ILI surveillance in one city/province and provided aggregated data over multiple seasons.

**Table 2:** Articles reporting the proportion of influenza B among all laboratory‐confirmed influenza

Author	Design	Study years	Patient population	Number tested for influenza	Number (n) (proportion, %) of N with laboratory‐confirmed influenza	Number (n) (proportion, %) of type B among all laboratory‐confirmed influenza
**Australia**						
Roche (2002)^117^	Passive‐laboratory‐based surveillance	1991‐2000	Laboratory reports (nationwide network)	340 730	16 805 (4.9, 4.6‐87.2 annually)	3614 (21.5, 12.8‐95.4 annually)
Teichtahl (1997)^118^	Case‐control study	Aug 1993‐July 1994	Hospitalized adults with asthma exacerbation	79	20 (25.2)	2 (10.0)
Moore (2009)^119^	Retrospective descriptive	May 1997‐Dec 2005	Specimens from children <18 years (1 hospital)	32 741 specimens	1951 (6.0)	304 (15.6)
Kelly (2000)^120^	Prospective laboratory‐supported surveillance	1998‐1999	Patients with ILI (17‐26 general practitioners)	152	65 (42.8)	3 (4.6)
Caini (2015)^34^	Prospective national surveillance of patients with ILI	2001‐2012	Laboratory‐confirmed influenza	179 137	179 137(100)	28 700 (16.0)
Druce (2005)^23^	Prospective hospital‐based surveillance	May‐Sept 2002 & 2003	Hospitalized patients with respiratory illness	3095	Only data by age	55 (1.8)
			Specimens collected from state‐wide influenza surveillance program	1159	‐	133 (11.5)
Fielding (2007)^25^	Prospective surveillance	May‐Oct 2006	Patients with ILI (74 general practitioners)	384	126 (32.8)	15 (11.9)
Iskander (2009)^24^	Prospective hospital‐based surveillance	June‐Oct 2006	Hospitalized children <5 years with ILI (1 hospital)	273	31 (11.4)	9 (29.0)
Lambert (2008)^26^	Prospective hospital‐based surveillance	July 2006‐Aug 2007	Children with ARI presenting to hospital (1 hospital)	303 specimens (295 patients)	37 (12.2)	7 (18.9)
Lester‐Smith (2009)^28^	Retrospective descriptive	Jan‐Dec 2007	Hospitalized children with influenza (1 hospital)	122	122 (100)	3 (2.5)
Miller (2008)^27^	Prospective surveillance	Apr‐Sept 2007	Patients with ILI (21 sentinel general practices)	403	189 (46.9)	24 (12.7)
			Notified laboratory‐confirmed influenza	1343	1343 (100)	186 (13.8)
Grant (2011)^121^	Prospective and retrospective surveillance	2010	Patients with ILI (32 general practices)	478	170 (35.6)	4 (2.4)
			Notifications of laboratory‐confirmed influenza to Health Department	1914	1914 (100)	88 (4.6)
Macesic (2013)^122^	Prospective hospital‐based surveillance	April‐Nov 2010 & 2011	Community‐acquired influenza (8‐15 hospitals)	572	572 (100)	58 (10.1)
			Nosocomial influenza	26	26 (100)	2 (7.7)
Levy (2014)^29^	Prospective surveillance	2010‐2012	Patients with ILI (31‐57 general practices)	448 (2010) 351 (2011) 1361 (2012)	146 (32.6) 84 (29.9) (2011) 603 (44.3) (2012)	56 (38.4) (2010) 18 (20.7) (2011) 259 (43) (2012)
Fielding (2013)^30^	Prospective surveillance	2012	Patients with ILI (41 general practices)	709	280 (39.5)	38 (13.6)
			Notified laboratory‐confirmed influenza	5058	5058 (100)	745 (14.7)
			Influenza complications network	389	389 (100)	50 (12.8)
Sullivan (2014)^123^	Prospective surveillance	2012	Patients with ILI (110 General practices nationally)	1414	593 (41.9)	106 (17.9)
Jennings (2015)^124^	Prospective surveillance	15 Jun‐12 Jul 2015	Laboratory‐confirmed influenza (13 laboratories in New South Wales)	1234	1234 (100)	821 (66.5)
		1 Apr‐18 Jul	Laboratory‐confirmed influenza presenting to Emergency Departments (4 hospitals)	88	88 (100)	41 (46.6)
WHO^55^	Prospective national surveillance	Jan‐Sept 2015	Laboratory‐confirmed influenza	Not given	‐	(61%)
			Laboratory‐confirmed influenza from general practices	2565	(30.9%)	(64%)
			Hospitalized laboratory‐confirmed influenza	‐	‐	(52%)
**Cambodia**						
Buecher (2010)^31^	Prospective hospital‐based surveillance	Feb‐May 2005‐2007	Patients with ILI during dry season (5 hospitals)	234	4 (1.7)	2 (50)
Mardy (2009)^32^	Prospective hospital‐ and clinic‐based surveillance	2006‐2008	Patients with ILI (5 outpatient departments) and ALRTI (2 hospitals) (2007‐08)	3148 (ILI) 1868 (ALRI)	338 (10.7) (5.8‐15.3 annually) 64 (3.4) (1.4‐3.6 annually)	ILI & ALRTI 148 (43.8) (34‐57.7 annually)
Kasper (2012)^125^	Prospective hospital‐based surveillance	Dec 2006‐Dec 2009	Acutely ill patients with fever (9 hospitals in South‐central Cambodia)	9968	1983 (19.9)	631 (31.8)
Saha (2014)^44^	Prospective clinic‐based surveillance	2006‐2011	Patients with ILI at sentinel centers	10 105	1574 (15.6) (7.6‐18.8 annually)	(22.9‐65.6) (annually)
Horm (2014)^33^	Prospective hospital‐based surveillance	2009‐2011	Patients with ILI (11 sites nationally) and ALRTI (2 hospitals)	7376 (ILI) 2248 (ALRI)	1262 (16.9) (14.5‐18.7 annually) 59 (2.6 (1.5‐3 annually)	ILI & ALRTI 515 (30.6) (12.6‐64.8 annually)
Timmermans (2016)^97^	Prospective surveillance	May 2010‐Dec 2012	Patients with ILI (4 sites in Western Cambodia)	586	168 (29.7)	76 (45.2)
**China**						
Cheng (2013)^35^	Prospective hospital‐ and clinic‐based surveillance (Shenzhen)	1995‐2009	Patients with URTI until 2003 (8 sites), ILI after 2003 (22‐31 sites)	25 377	2678 (10.6) (0.2‐25 annually)	757 (28.3) (2‐79 annually)
Tang (2008)^126^	Prospective hospital‐based descriptive (Gangzhou)	Jan‐2001‐Dec 2006	Hospitalized children <14 years with ALRI	34 885	760 (2.2)	72 (9.5)
Lin (2013)^36^	Prospective hospital and laboratory‐based surveillance (Guangdong)	Jan 2004‐Dec 2012	Patients with ILI (14 hospitals)	107 115	17 454 (16.3)	4978 (28.5)
			ILI outbreak surveillance	919 outbreaks	828 outbreaks (90.1)	Multiple B outbreaks in 2005, 2007, 2010, 2012
Caini (2015)^34^	Prospective national ILI surveillance	2006‐2012	Laboratory‐confirmed influenza (South)	122 215	122 215 (100)	35 910 (29.4)
		2005‐2011	(North)	64 306	64 306 (100)	19 956 (31.0)
Yang (2012)^127^	Prospective outpatient surveillance (Beijing)	May 2006‐Jan 2010	Patients ≥ 14 years with ARI (1 hospital)	7776	1854 (23.8)	405 (21.8)
Ji (2010)^128^	Retrospective hospital‐based descriptive (Suzhou)	Jan 2007‐Dec 2008	Hospitalized children <5 years respiratory infection (1 hospital)	7789	120 (1.5)	25 (20.8)
Yang (2009)^37^	Prospective outpatient and ED surveillance (Beijing)	Sept 2007‐Apr 2008	Patients with ILI (14 hospitals)	2057	611 (29.7)	450 (73.6)
Guo (2012)^129^	Prospective outpatient surveillance (Zhuhai City)	2008	Patients with ILI (28 hospitals)	1485	135 (9.1)	(23.7)
		2009		2144	604 (28.2)	(9.1)
Peng (2012)^130^	Prospective hospital‐based surveillance (Wuhan)	Jul 2008‐Jun 2010	Children ≤ 14 years with ILI (1 hospital)	1472	455 (30.9)	100 (22.0)
Ge (2012)^38^	Prospective outpatient surveillance (Shanghai)	Jun 2009‐May 2011	Children with ILI (1 hospital)	2356	608 (25.8)	142 (23.4)
Lu (2013)^131^	Prospective descriptive (Jinan)	Aug 2009‐Sept 2010	Patients ≥ 14 years with ARI (1 hospital)	596	124 (20.8)	75 (60.5)
Wei (2013)^40^	Prospective hospital‐based surveillance	Apr 2009‐Mar 2011	Patients with ILI	6143	1645 (26.8)	348 (21.2)
Zhu (2013)^132^	Laboratory‐based descriptive (Hubei & Zhejiang)	2009‐2010	Patients with respiratory infection (inpatients and outpatients)	341	54 (15.8)	18 (33.3)
Zhao (2015)^41^	Prospective outpatient surveillance (Shanghai)	2009‐2014	Patients with ILI	71 354	19 974 (28.0)	6688 (33.5)
Li (2013)^133^	Prospective outpatient surveillance (Zhuhai City)	Jan‐Dec 2010	Patients with ILI (1 hospital)	924	187 (20.2)	96 (51.3)
Yu (2013)^39^	Prospective hospital‐based surveillance (Jingzhou City)	2010‐2012	Hospitalized patients with SARI (4 hospitals)	16 208	2057 (12.7)	998 (48.5)
Huo (2012)^134^	Prospective outpatient surveillance (Nanjing)	Nov 2010‐ Oct 2011	Patients with ILI (2 hospitals, 1 laboratory)	486	178 (36.3)	37 (20.8)
Chen (2014)^135^	Prospective hospital‐based surveillance (Changsha)	2010‐2012	Patients with ILI (2 hospitals)	2955	278 (9.4)	83 (29.9)
Wang (2014)^136^	Retrospective modeling study (Gangzhou)	Jan 2010‐Dec 2012	Patients with ILI	8258	1081 (13.1)	360 (33.3)
Yu (2012)^137^	Prospective hospital‐based descriptive (Beijing)	May 2010‐Apr 2011	Patients ≥ 14 years in ED with ARI (1 hospital)	416	70 (16.8)	3 (4.3)
Fu (2015)^138^	Prospective clinic‐based surveillance (Shanghai)	Jan 2011‐Dec 2013	Patients with ILI (2 hospitals)	1970	392 (19.9)	162 (41.3)
Ju (2014)^139^	Prospective hospital descriptive (Huizhou)	Jul 2011‐Jul 2013	Hospitalized patients with ILI (1 hospital)	1046	209 (20.0)	74 (35.4)
Wang (2016)^140^	Prospective hospital‐based surveillance (Suzhou))	Apr 2011‐Mar 2014	Children <5 years with ILI presenting to outpatient or ED	3662	619 (16.9)	349 (56.4)
**Indonesia**						
Beckett (2004)^42^	Laboratory‐based surveillance	Aug 1999‐Jan 2003	Children >4 and adults with ILI at 6 sentinel centers	1372	130 (9.5)	33 (25.4)
Kosasih (2013)^43^	Prospective health center and hospital‐based Surveillance	Jan 2003‐Dec 2007	Inpatients and outpatients with ILI at 5 (2003) to 48 (2006‐07) sentinel centers	21 030	4236 (20.1)	1487 (35.1)
Caini (2015)^34^	Prospective national surveillance of patients with ILI	2003‐2007	Laboratory‐confirmed influenza	3653	3653 (100)	1314 (36.0)
Saha (2014)^44^	Prospective clinic‐based surveillance	2010‐2011	Patients with ILI at sentinel centers	15 150 specimens	2511 (16.6)	(49.9 in 2010, 24.8 in 2011)
Storms (2015)^45^	Enhanced prospective surveillance in East Jakarta	Oct 2011‐Sept 2012	Patients with ILI at 4 outpatient clinics	3278	1131 (34.5)	536 (47.4)
			Patients with SARI at 6 hospitals	1787	276 (15.4)	132 (47.8)
**Laos**						
Vongphrachanh (2010)^47^	Prospective hospital‐based surveillance	Jan 2007‐Dec 2008	Patients with ILI presenting to hospital OPD/ED (3 hospitals)	526	155 (29.5)	92 (59.3)
		Aug‐Dec 2008	Hospitalized ALRTI	79	10 (12.7)	4 (40.0)
Khamphaphongphane (2013)^46^	Laboratory‐based surveillance	Jan 2008‐ Dec 2010	Patients with ILI presenting to hospital OPD/ED (7 hospitals)	2338 specimens	523 (22.4) (20.9‐23 annually)	142 (27.7) (2.7‐66.7 annually)
Saha (2014)^44^	Prospective clinic‐based surveillance	2006‐2011	Patients with ILI at sentinel centers	5949	1302 (21.9) (12.8‐29.8 annually)	(2.2‐56.5 annually)
Sentilhes (2013)^48^	Prospective hospital‐based surveillance	Aug 2009‐Oct 2010	Patients hospitalized with ALRTI	292 specimens	23 (7.9)	5 (21.8)
**Malaysia**						
Chan (1999)^141^	Retrospective descriptive	Jan 1982‐Dec 1997	Children <24 months with LTRI	5697	77 (1.4)	18 (23.4)
Khor (2012)^81^	Retrospective hospital‐based descriptive	1982‐2008	Samples from hospitalized children ≤5 years (1 hospital)	10 269	297 (2.9)	64 (21.9)
Sam (2015)^142^	Laboratory‐based surveillance	1995‐2008	Laboratory‐confirmed influenza 1 month to 49 years	338	338 (100)	88 (26.0)
Sam (2010)^143^	Retrospective descriptive	2002‐2007	Hospitalized children <15 years with laboratory‐confirmed influenza	132	132 (100)	35 (26.5)
Saat (2010)^49^	Prospective laboratory‐based surveillance	Jan 2005‐Dec 2009	Patients with ILI (nationwide)	7117 specimens	993 (14.0) (10.2‐31.6 annually)	305 (30.7) (18‐51 annually)
Saha (2014)^44^	Prospective clinic‐based surveillance	2006‐2011	Patients with ILI at sentinel centers	10 323	894 (8.7) (3.1‐13.4 annually)	(20.2‐62.6, 2006‐2010)
**Myanmar**						
Hasegawa (2006)^50^	Prospective hospital‐ and clinic‐based surveillance	Sept 2003‐Dec 2004	Patients with ILI (1 hospital, 2 general practitioners)	616	139 (22.6)	6 (4.3)
Hasegawa (2006)^51^	Prospective hospital‐ and clinic‐based surveillance	2005	Patients with ILI (1 hospital, 2 general practitioners)	992	268 (27.0)	125 (46.6)
Dapat (2009)^52^	Prospective hospital‐ and clinic‐based surveillance	2005‐2007	Patients with ILI (1 hospital, 1 clinic)	2618	522 (19.9) (24 in 2005, 11.4 in 2006, 19 in 2007)	267 (51.1) (42 in 2005, 0 in 2006, 67 in 2007)
**New Zealand**						
Huang (2008)^53^	Prospective national surveillance	1997‐2006	All influenza diagnoses sentinel general practice, laboratory, hospital, and mortality surveillance	‐	Average 718 annually	(0‐92 annually)
Laing (2001)^144^	Prospective hospital‐based surveillance	Jul 1999‐Jul 2000	Hospitalized adults >18 with CAP	474	39 (8.2)	8 (20.5)
Caini (2015)^34^	Prospective national surveillance of patients with ILI	2000‐2012	Laboratory‐confirmed influenza	17 629	17 629 (100)	2965 (16.8)
Jennings (2004)^145^	Prospective hospital‐based descriptive	Jul‐Nov 2001	Children with ARI (1 hospital)	75	10 (13.3)	7 (70.0)
Huang (2007)^54^	Prospective national surveillance	2005	Laboratory‐confirmed influenza sentinel general practice, laboratory and hospital‐based surveillance	845	845 (100)	734 (86.9)
Turner (2014)^146^	Prospective hospital‐ and clinic‐based surveillance	Apr‐Sept 2013	Patients with ILI (18 sentinel general practices) and SARI (4 hospitals)	1298 (ILI) 886 (SARI)	182 (21) 391 (30)	ILI&SARI 221 (39)
WHO^55^	Sentinel ILI & SARI and laboratory surveillance	Jan‐Sept 2015	Laboratory‐confirmed influenza	Not given	5235 (100)	680 (13%)
			Patients with ILI SARI patients	13891206	614 (44.2%)285 (23.6)	308 (50%)Not mentioned
**Philippines**						
Saha (2014)^44^	Prospective clinic‐based surveillance	2006‐2011	Patients with ILI at sentinel centers	69 108	12 607 (18.2) (6.9‐33.1 annually)	(Range 2.3‐75.7)
Suzuki (2012)^147^	Prospective descriptive	May 2008‐May 2009	Hospitalized children 8 day‐13 years with severe CAP (1 center)	819	29 (3.5)	11 (37.9)
Tallo (2014)^60^	Prospective clinic‐based surveillance	Jan 2009‐Dec 2011	Patients with ILI and SARI at sentinel centers	5915 (ILI) 2659 (SARI)	1282 (21.7) (12.3‐25.6 annually) 226 (8.5) (6‐11.2 annually)	397 (31.0) (6‐61.2 annually) 72 (31.9) (11.4‐49.4 annually)
Otomaru (2015)^61^	Prospective clinic‐based surveillance	Jan 2010‐Mar 2013	Patients with ILI at sentinel centers	2031	225 (11.1) (3.2‐16.0 annually)	104 (46.2) (23.8‐81.3 annually)
**PNG**						
Kono (2014)^56^	Prospective surveillance	2010	Patients with ILI (2 hospitals)	300	88 (29.3)	38 (43.2)
**Singapore**						
Chew (1998)^82^	Retrospective descriptive	Sept 1990‐Sept 1994	Patients tested for respiratory pathogens (2 hospitals)	12 354 specimens	426 (3.4)	92 (21.6)
Chow (2006)^62^	Retrospective surveillance	Jan 1996‐ Dec 2003	Samples from outpatients or inpatients with ILI (National Influenza Centre)	57 060 specimens	3829 (6.7)	333 (13.9)
Yang (2011)^63^	Retrospective surveillance	2004‐2006	Samples from outpatients or inpatients with ILI (Ministry of Health)	29 329 specimens	1291 (5.5)	305 (23.6) (16.3‐33.0 annually)
Seah (2010)^68^	Prospective descriptive	Mar 2006‐Apr 2007	Military personnel with febrile respiratory illness (1 camp)	1354 specimens	489 (36.1)	159 (32.5)
Virk (2014)^65^	Prospective surveillance	May‐Oct 2007	Students and staff with ILI (National University of Singapore)	266	56 (21.1)	9 (16.1)
Tan (2015)^64^	Prospective surveillance	2007‐2009	Students and staff with ILI (National University of Singapore)	500	164 (32.8)	11 (6.7)
Saha (2014)^44^	Prospective clinic‐based surveillance	2007‐2011	Patients with ILI at sentinel centers	55 449	12 801 (23.1)	(2.7‐79.1 2007‐2011)
Caini (2015)^34^	Prospective national surveillance of patients with ILI	2007‐2012	Laboratory‐confirmed influenza	12 001	12 001 (100)	2311 (19.3)
Yap (2012)^67^	Prospective descriptive	May 2009‐June 2010	Military personnel with febrile respiratory illness (4 camps)	2858	821 (28.7)	269 (32.8)
Tan (2014)^66^	Prospective surveillance	May 2009‐Oct 2012	Military personnel with febrile respiratory illness (5 camps)	7733	972 (12.6)	449 (46.2)
**South Korea**						
Yun (1995)^78^	Prospective hospital‐based descriptive	Nov 1990‐Apr 1994	Children with ALRI + children visiting the OPD or with nosocomial ALRI	804 specimens (712 patients)	42 (5.2)	11 (26.2)
Lee (2007)^148^	Prospective clinical & laboratory‐based surveillance	Sept 2000‐Oct 2001	Patients with ILI	2972	144 (4.8)	0 (0)
Kim (2008)^77^	Retrospective descriptive	Mar 2004‐Dec 2005	Hospitalized children <15 years with LRTI with NPA	400	76 (19)	32 (42.1)
Seo (2014)^69^	Retrospective laboratory‐based descriptive	Jan 2005‐Dec 2008	Children <19 years with ARI	21 641 specimens	1116 (5.2)	484 (43.4)
			Adults with ARI	2165 specimens	217 (10.0)	121 (55.8)
Choi (2012)^149^	Prospective descriptive	Mar 2010‐Feb 2011	Adults ≥ 18 year in ICU with severe CAP or HCAP	198	12 (6.1)	1 (8.3)
Noh (2013)^150^	Prospective hospital‐based surveillance	Sept 2011‐Jun 2012	Adults ≥ 18 years who visited an ED with ILI	1983	846 (42.7)	169 (20.0)
Song (2013)^76^	Prospective ED‐based surveillance	Oct 2011‐Sept 2012	Laboratory‐confirmed influenza	7213	7213 (100)	3217 (44.6)
Wie (2013)^151^	Prospective ED‐based surveillance	Oct 2011‐May 2012	Adults with ILI	2129	850 (39.9)	194 (22.8)
Seo (2014)^152^	Prospective ED‐based surveillance	Oct‐2011‐June 2012	Patients with ILI at ED	4490 tested	Not given	Max 58% of weekly samples
Choi (2015)^153^	Retrospective case control	Sept 2011‐May 2012	Patients visiting hospital with ILI	7390 tested	1130 (15.3)	452 (40)
Ahn (2015)^154^	Retrospective laboratory‐based descriptive	Jan 2012‐Apr 2013	Adults >16 tested for respiratory viruses	291 specimens (282 patients)	47 (16.1)	4 (8.5)
**Taiwan**						
Lin (2004)^80^	Prospective hospital‐based descriptive	Aug 1995‐July 1997	Pediatric outpatients with URTI	910	112 (12.3)	58 (51.8)
Tsai (2001)^79^	Prospective hospital and clinic‐based Surveillance	Jan 1997‐Dec 1999	Children <12 years with RTI (inpatients and outpatients)	6986	565 (8.1)	181 (32.0)
Huang (2009)^155^	Retrospective descriptive	Jan 1997‐May 2007	Laboratory‐confirmed influenza	2651	2651 (100)	1168 (44.1)
Hu (2004)^156^	Retrospective descriptive	Jan 2000‐Dec 2001	Children with laboratory‐confirmed influenza	197	197 (100)	124 (62.9)
Shih (2005)^157^	Prospective laboratory surveillance	Oct 2000‐Mar 2004	Patients with suspected RTI (inpatients and outpatients)	32 775	3244 (9.9)	1,2.75 (39.3)
Chi (2008)^158^	Retrospective hospital‐based descriptive	Jan 2001‐Dec 2006	Children with LRTI (inpatients and outpatients)	20 405 specimens	745 (3.7)	118 (15.8)
Jian (2008)^159^	Prospective laboratory surveillance	2003‐2006	Patients with suspected RTI (inpatients and outpatients)	34 312	4007 (11.7)	1336 (33.3)
Lin (2013)^160^	Prospective laboratory and sentinel physician‐based surveillance	2003‐2007	Patients with URTI or LRTI symptoms	12 190	1150 (9.4)	651 (56.6)
Jian (2008)^161^	Prospective laboratory surveillance	2004‐2005	Laboratory‐confirmed influenza	1183	1183 (100)	971 (82.1)
		2006‐2007		1534	1534 (100)	1219 (79.5)
Wang (2009)^162^	Retrospective and prospective descriptive	Nov 2006‐Feb 2007	Children <18 years with ILI (inpatients, ED, outpatients)	198 specimens (196 children)	101 (51.0)	87 (86.1)
Chen (2012)^70^	Prospective hospital‐based descriptive	Jan 2009‐March 2011	Children <24 months hospitalized with bronchiolitis	113	5 (4.4)	0 (0)
Chuang (2012)^112^	Prospective national surveillance	2009‐2010	Samples from patients with ARTI	14 788 specimens	3970 (26.9)	545 (13.7)
		2010‐2011	Samples from patients with ARTI	11 813 specimens	2767 (23.4)	489 (17.7)
		2009‐2010	Patients with influenza hospitalized with	1297	1297 (100)	82 (6.3)
		2010‐2011	Pulmonary complications	1751	1751 (100)	50 (2.9)
Lo (2013)^71^	Prospective national surveillance	Jun 2011‐Jun 2012	Outpatients with ILI	14 943	3285 (22.0)	2382 (72.5)
			Suspected influenza with complications	2675	1704 (63.7)	1034 (60.7)
**Thailand**						
Suzuki (1997)^163^	Prospective surveillance	Each August 1991‐1994	Patients with suspected influenza (2 hospitals)	186 specimens	32 (17.2)	11 (34.4)
Sirivichayakul (2000)^164^	Prospective descriptive	June 1998‐May 1999	Nursing students with ILI (University)	106	35 (33.0)	2 (5.7)
Thawatsupha (2000)^165^	Prospective hospital‐based surveillance	Jan‐Dec 2001	Outpatients with ARI (6 hospitals)	711 specimens	338 (54.6)	102 (30.2)
Olsen (2010)^84^	Prospective hospital‐based surveillance	Sept 2003‐Dec 2005	Hospitalized patients with acute LRTI (all hospitals in 2 provinces)	3910	586 (15.0)	150 (25.6)
Suntarattiwong (2007)^166^	Prospective descriptive hospital‐based study	July 2004‐July 2005	Hospitalized children 0‐5 years with LRTI (1 hospital)	456	39 (8.6)	5 (12.8)
Chittaganpitch (2011)^72^	Prospective surveillance	2004‐2010	Patients with ILI at 11 sentinel centers,	19 121	3896 (20.4) (15‐25 annually^a^)	1284 (33.0 range 13‐43^a^)
		Sep‐Dec 2010	Inpatients with SARI at 3 hospitals	336	71 (21.1)	20 (28.2)
Simmerman (2009)^74^	Prospective hospital‐based surveillance	Jan 2005‐Dec 2008	Hospitalized patients with pneumonia (all hospitals in 2 provinces)	13 119	1391 (10.6) (4.1‐16.0 annually)	444 (31.9) (range 13.6‐44.9 per year)
Hara (2011)^167^	Hospital‐based descriptive	2006‐2008	Hospitalized patients with CAP (hospital and HIV center)	119	7 (5.9)	1 (14.3)
Saha (2014)^44^	Prospective clinic‐based surveillance	2007‐2011	Patients with ILI at sentinel centers	17 421	3802 (21.8) (18.4‐25.5 annually)	(12.9‐42.9)
Baggett (2012)^83^	Prospective active, population‐based Surveillance	Jan 2009‐Dec 2010	Hospitalized patients with acute LRTI (all hospitals in 2 provinces)	7207	902 (12.5)	120 (13.3)
Prachayangprecha (2013)^168^	Prospective hospital‐based surveillance	Jun 2009‐Jul 2012	Patients with ILI attending hospitals in Bangkok	6050	2969 (49.0)	3% of tested samples
Dawood (2014)^114^	Prospective hospital‐based surveillance	July‐Dec 2010, 2011	Hospitalized patients with ARI (all hospitals in 2 provinces)	1545	279 (18.1)	32 (11.5)
Tewawong (2015)^73^	Laboratory‐based surveillance	Jan 2010‐Feb 2014	Samples from patients with ILI (3 provinces)	14 418 specimens	3050 (21.2)	471 (15.4)
**Vietnam**	Prospective laboratory‐based surveillance					
Nguyen (2007)^57^		2001‐2003	Outpatients with ILI (12 centers)	4708	119 (2.5)	59 (49.6) (range 0‐77)
Do (2011)^169^	Prospective hospital‐based descriptive	Nov 2004‐Jan 2008	Hospitalized children <15 years with ARI (1 center)	309	51 (16.5)	24 (47.1)
Nguyen (2009)^58^	Prospective hospital and clinic‐based surveillance	Jan 2006‐Dec 2007	Outpatients with ILI (15 clinics)	11 082	2112 (19.1)	585 (27.7)
Nguyen (2013)^59^	Prospective surveillance	Jan 2006‐ Dec 2010	Outpatients with ILI (7‐15 centers)	29 804	6616 (21.9) (18‐26 annually)	2163 (33.2) (23.3‐51.6 annually)
Saha (2014)^44^	Prospective clinic‐based surveillance	2006‐2011	Patients with ILI at sentinel centers	29 499	5241 (17.8)	(Range 0‐41.3.8)
Caini (2015)^34^	Prospective national surveillance of patients with ILI	2006‐2013	Laboratory‐confirmed influenza	8647	8647 (100)	3011 (34.8)
Takahasi (2013)^75^	Prospective descriptive	Sept 2009‐Aug 2010	Hospitalized patients ≥ 15 years with LRTI (1 center)	323	45 (13.9)	13 (28.9)
**WHO Western Pacific region**						
14 countries^170^	Regional ILI and laboratory surveillance	2006	Data from national influenza centers (patients with ILI)	65 103	7425 (11.4)	3032 (40.8)
		2007		92 939	11 143 (12.0)	3846 (34.5)
		2008		94 274	11 025 (11.7)	3599 (32.6)
		2009		366 164	115 554 (31.6)	4886 (4.2)
		2010		307 584	51 573 (16.8)	25 565 (49.6)

A(L)RTI, acute (lower) respiratory tract infection; CAP, community‐acquired pneumonia; ED, emergency department; GP, general practice; HCAP, healthcare‐associated pneumonia; HIV, human immunodeficiency virus; ICU, intensive care unit; ILI, influenza‐like illness; LRTI, lower respiratory tract infections; NPA, nasopharyngeal aspirate; OPD, outpatient department; PNG, Papua New Guinea; P&I, pneumonia and influenza; RTI, respiratory tract infection; SARI, severe acute respiratory illness.

a Percentage from 2004 data not taken as only 11 subjects included in the 2004 surveillance.

A study in Shenzhen in the southern province of Guangdong from 1995 to 2009 reported an annual influenza detection rate among ILI cases ranging from 0.2% in 1998 to 25% in 2009. The lowest proportion of influenza B among all influenza‐positive samples was in 2008.^35^ Influenza B predominated in 4 of the 15 study years with the peak (79%) reported in 1997. Multiple influenza B outbreaks between 2004 and 2012 were reported in Guangdong province, including in 2010 following the peak of A/H1N1/pdm09.^36^ The 2010 influenza B peak was also reported in studies conducted in northern, central, and eastern part of China.^37^, ^38^, ^39^, ^40^

A study conducted in central China assessed SARI hospitalizations by type of influenza and found that in 2010‐11, A (H3N2) virus was associated with a higher SARI hospitalization rate (55/100 000) than both influenza B and A (H1N1)pdm2009. In the following year, the incidence of SARI hospitalization was highest with influenza B (98/100 000).^39^

Between the 2009‐14 seasons, on average, 45% of circulating B lineages in Shanghai differed antigenically from the vaccine strain. During the period from 2009 to 2012, B/Victoria lineage matching the seasonal influenza vaccines strain predominated over the B/Yamagata lineage. The proportion of B/Yamagata lineage exceeded 97% of all circulating influenza B viruses in late 2012 and resulted in complete vaccine B strain mismatch.^41^

#### 3.2.4 Indonesia

We identified five articles with data relevant to Indonesia (Table 2). Three of these papers were based on ILI surveillance of multiple seasons, reporting a proportion of type B viruses between 25.4% and 36% among all influenza‐positive samples during the overall study period.^34^, ^42^, ^43^ Two papers provided season‐specific information on the proportion of influenza B among all ILI cases with laboratory‐confirmed influenza in 2010‐11 and 2011‐12.^44^, ^45^ One of them also provided data in SARI patients with laboratory‐confirmed influenza in 2011‐12. In that study, the influenza detection rate was higher among ILI than among SARI patients (34.5% vs 15.4%). In both patient groups, 47% of laboratory‐confirmed influenza cases were influenza B.^45^

#### 3.2.5 Laos

ILI virological surveillance started in Lao People's Democratic Republic (PDR) in 2007. We identified four articles assessing influenza in Laos during the period from 2007 to 2011. One study based on ILI surveillance during the period 2008‐10 reported influenza‐positivity rate of 20.9%‐23% in the three years. The proportion of influenza B among all influenza‐positive samples dropped from 66.7% in 2008 to 2.7% in 2009 when A/H3N2 and A/H1N1pdm09 became predominant, then increased to 33.7% in 2010.^46^

Two studies assessing influenza among patients hospitalized with ALRI showed that the influenza‐positivity rate (12.7%) and influenza B proportion among ALRI cases (40%) with laboratory‐confirmed influenza in 2008 were higher comparing to the findings in 2009‐10 (7.9% and 21.8%, respectively).^47^, ^48^

#### 3.2.6 Malaysia

Six articles were identified for Malaysia reporting studies conducted between 1982 and 2011, of which four reported aggregated data across multiple seasons.

From 2005‐2009, national ILI surveillance detected influenza in 10.2% (2008) to 31.6% (2006) of ILI cases.^49^ In this period, the proportion of influenza B was lowest in 2008 (18%) and highest in 2005 (51%). Peak season of influenza fell between May and August. Complete influenza B strain mismatch with Southern Hemisphere vaccine B strain was observed in 2005 and 2009 and significant mismatch in 2007 (Table 5).

Laboratory‐based surveillance of ILI patients presenting to sentinel centers showed that the percentage of samples tested positive for influenza ranged between 3.1 and 13.4% in 2006‐11.^44^, ^49^ The percentage of B strains among the influenza‐positive samples was 30.7% on average from 2005 to 2009, and 20.2% to 62.6% annually between 2006 and 2010.

#### 3.2.7 Myanmar

Three articles reported the results of sentinel site‐based surveillance study in Myanmar between 2003 and 2007.^50^, ^51^, ^52^ The proportion of influenza‐positive samples obtained from patients with ILI ranged from 11.4% in 2006 to 24%‐27.0% in 2005. Few influenza B cases were detected in 2003‐04 and 2006. The proportion of influenza‐positive samples that were type B ranged from 42%‐46.6% in 2005 with a majority of influenza B isolates belonging to the Victoria lineage. In 2007, 67% of influenza viruses were influenza B and all belonged to the B/Victoria lineage.^50^, ^51^, ^52^

#### 3.2.8 New Zealand

Seven articles describing influenza in New Zealand between 1990 and 2015 were identified. National influenza surveillance data showed that during 1997‐2008, on average 718 cases of laboratory‐confirmed influenza were detected every year.^53^ Influenza surveillance in 2005 recorded the highest influenza B activity since 1990 with co‐circulation of influenza strains from B/Victoria and B/Yamagata lineages, which resulted in significant B strain mismatch with the recommended vaccine composition.^54^ In 2015, 44.2% of patients with ILI and 23.6% SARI patients tested positive for influenza.^55^ The proportion of influenza B among all influenza‐positive samples was 13%, but half of them mismatched the B strain contained in the influenza vaccines for that season (Table 3).

**Table 3:** Circulating and vaccine influenza type B lineages in northern and Southern Hemisphere temperate/subtropical countries

Year	Vaccine lineage	% type B	Circulating lineage (%)		Degree of mismatch	% type B	Circulating lineage (%)		Degree of mismatch	% type B	Circulating lineage (%)		Degree of mismatch
			Victoria	Yamagata			Victoria	Yamagata			Victoria	Yamagata	
Northern Hemisphere countries		South Korea (surveillance^171^)				Taiwan (surveillance^172^)				China (clinical samples^41^, surveillance^90^)			
2007‐08	Victoria	64.1	0	100	Complete	‐	‐	‐	‐	73.6	‐	Dominant	‐
2008‐09	Yamagata	1.2	Reported	Reported	‐	‐	‐	‐	‐	‐	74	26	Significant
2009‐10	Victoria	26.4	100	0	‐	32	91	9	‐	‐	‐	‐	‐
2010‐11	Victoria	0.9	Reported	Reported	‐	21	83	17	‐	‐	‐	‐	‐
2011‐12	Victoria	48.5	73	27	Partial	76	14	86	Significant	‐	‐	‐	‐
2012‐13	Yamagata	5.6	65	35	Significant	2	13	87	‐	‐	97	3	Complete
2013‐14	Yamagata	53	86	14	Significant	25	81	19	Significant	‐	‐	97	‐
2014‐15	Yamagata	37	‐	‐	‐	26	55	45	Significant	‐	‐	98	‐

Southern Hemisphere countries		Australia (surveillance^20^, ^21^, ^22^)				New Zealand (surveillance^55^, ^173^)			
2005	Yamagata	23	51	49	Significant	87	82	18	Significant
2006	Victoria	35	94	6	‐	1	60	40	Significant
2007	Victoria	7	21	79	Significant	23	1	99	Complete
2008	Yamagata	67	51	49	Significant	58	77	23	Significant
2009	Yamagata	1	62	38	Significant	0	0	0	‐
2010	Victoria	7	91	9	‐	1	100	0	‐
2011	Victoria	38	98	2	‐	50	98	2	‐
2012	Victoria	33	89	11	‐	14	16	84	Significant
2013	Yamagata	37	7	93	‐	40	1	99	‐
2014	Yamagata	4	10	90	‐	11	4	96	‐
2015	Yamagata	62	36	64	Partial	51	48	52	Significant

Shading indicates mismatch years. Partial mismatch: between 20%‐<40% of circulating influenza type B strain was not the vaccine strain; significant mismatch ≥40%; complete mismatch ≥95%; ‐: no data, or not a mismatch year. Degree of mismatch not categorized for countries/seasons without numerical lineage distribution estimates.

#### 3.2.9 Papua New Guinea

In the one study identified as providing information on influenza in Papua New Guinea, 29.3% of samples from patients with ILI received by the Papua New Guinea National Influenza Centre in 2010 had laboratory‐confirmed influenza, of which 43.2% were influenza type B.^56^

#### 3.2.10 Philippines

Four articles provided information on influenza type B in the Philippines. Three articles reported clinic‐based surveillance of patients with ILI in different regions during an overlapping period from 2006 to 2013. The largest study in 2006‐11 collected 69 108 specimens, and the influenza virus detection rate varied between 6.9% (in 2008) and 33.1% (2009).^44^, ^57^, ^58^, ^59^ Influenza B was the predominant type circulating in 2008 (75.7%) as well as in 2010 (50.1%).

Surveillance in Baguio city in the North and in the Eastern Visayas region during 2010‐11 showed that regional circulation of influenza viruses and predominant types varied in the same season. Influenza detection rate among ILI patients and the proportion of influenza B among all influenza‐positive samples in Baguio city (25.6% and 61.2%, respectively) were higher than in Eastern Visayas region (16% and 54%) in 2010 but lower in 2011 (12.3% and 22% in Baguio city; 14.6% and 37% in Eastern Visayas region, respectively).^60^, ^61^ Surveillance in Baguio city also assessed influenza among SARI patients. In all three study years, influenza detection rates were lower among SARI patients than patients with ILI, while the proportion of influenza B was higher among SARI patients with laboratory‐confirmed influenza than ILI patients with laboratory‐confirmed influenza in 2009 and 2011.^60^

#### 3.2.11 Singapore

In Singapore, the National Influenza Centre carries out influenza virus surveillance using samples from public hospitals and sentinel clinics.^62^ Ten articles reporting data in Singapore from 1990 until 2012 were identified (Table 2). Four articles report the results of the national surveillance of samples from inpatients and outpatients presenting to sentinel centers with ILI between 1996 and 2012. The percentage of all samples with laboratory‐confirmed influenza ranged from 2.5% in 2007 to 50.4% in 2010.^34^, ^44^, ^62^, ^63^ The proportion of influenza‐positive samples that were influenza B was lowest in 2009 (2.7%) and highest in 2007 (79.1%).

Several studies reported influenza detection rates and proportions of influenza B viruses in various seasons among specific groups. Among students and staff at the National University of Singapore presenting with ILI, 21.1% had laboratory‐confirmed influenza in 2007 and 32.8% in 2007‐09. Although the study conducted in 2007‐09 detected a higher influenza‐positivity rate, the proportion of influenza B was lower comparing to the study in 2007 (6.7% vs 16.1%) as influenza A/H1N1pdm09 became the predominant type in the second half of the 2007‐09 study.^64^, ^65^

Three studies evaluated influenza in military personnel with febrile respiratory illness in 2006‐07, 2009‐10, and 2009‐12.^66^, ^67^, ^68^ Influenza detection rate was 36.1% and 28.7% in the two earlier seasons, both with the proportion of influenza B at 33%. Season‐specific influenza positivity and the proportion of influenza B after 2010 were not reported.

#### 3.2.12 South Korea

Eleven articles reported the distribution of influenza A and B viruses in South Korea between 1990 and 2013 (Table 2). Six studies were conducted in hospital emergency or outpatient departments, one included hospitalized children, one included adults in the intensive care unit (ICU), and three studies reported laboratory surveillance. Between 2007 and 2015, the Korean Influenza Surveillance System reported three influenza seasons with around 50% or higher proportion of influenza B; all three seasons had B lineage mismatch of >20% (Table 3). The highest percentage of influenza B positive samples was reported in a retrospective laboratory‐based study in which 43.4% of children and 55.8% of adults with laboratory‐confirmed influenza had type B.^69^ The study period encompassed the 2007‐08 influenza season, in which 64.1% of circulating influenza viruses were type B strains with a complete vaccine mismatch (Table 3).

#### 3.2.13 Taiwan

Thirteen studies conducted between 1995 and 2012 were identified for Taiwan (Table 2). Five articles reported data in children, and no studies specifically reported on influenza B in adults. All studies conducted before 2009 detected influenza B from the study population, but information on B lineages was not reported. The proportion of influenza‐positive samples that were type B varied across seasons, age groups, and diagnoses. One study conducted in 2009‐11 with low level of B strain mismatch found that in both seasons, the proportion of influenza B among all laboratory‐confirmed influenza cases was lower among patients hospitalized with pulmonary complications of influenza (2.9%‐6.3%) comparing to patients with acute respiratory tract infection (ARTI, 13.7%‐17.7%).^70^ In the following season (2011‐12) when influenza B was predominant and the circulating B strain significantly mismatched the vaccine strain, one study reported a significantly higher proportion of influenza B in both ILI patients with laboratory‐confirmed influenza (72.5%) and patients of suspected influenza with complications (60.7%), suggesting that the high proportion of influenza B together with the significant B strain mismatch (86%) resulted in heavy morbidity in that season.^71^

National Influenza Surveillance by the Taiwan CDC showed that since 2009, in all but one season, the proportion of influenza B viruses was >20% among all influenza viruses detected, including in 2009 during the A/H1N1/pdm09 pandemic. Significant influenza B mismatches were observed in three seasons, especially in 2011‐12 when 76% of the circulating influenza viruses were type B (Table 3).

#### 3.2.14 Thailand

Influenza sentinel surveillance throughout Thailand during 2005‐10 found that 15‐25% of samples from ILI patients tested positive for influenza. The highest proportion of influenza B among all laboratory‐confirmed influenza patients was 40% in 2007, while the lowest was 13% in 2009.^72^

Thirteen relevant articles were identified for Thailand that reported for seasons between 1998 and 2014. One large laboratory‐based surveillance study detected influenza viruses in 18.4%‐25.5% of samples collected from patients with ILI between 2007 and 2011.^44^, ^73^ The percentages of influenza‐positive samples that were influenza B ranged from 12.9% in 2009 to 42.9% in 2008.^73^

Seven articles included hospitalized patients with RTIs, pneumonia, or SARI from 2003 to 2011. The percentage of clinical specimens with laboratory‐confirmed influenza varied from 4.1% among patients hospitalized with pneumonia in 2006 to 21.1% among hospitalized SARI patients in 2010. Among hospitalized patients, the highest proportion (45%) of laboratory‐confirmed influenza cases that were influenza B was seen in 2008, when circulation of influenza B predominated but with significant mismatch with the influenza B strain included in the Southern Hemisphere vaccine.^74^

#### 3.2.15 Vietnam

National influenza surveillance using sentinel sites across Vietnam reported influenza detection rates among ILI patients between 18% and 26% during 2006‐10. In this period, the lowest proportion of influenza B among all laboratory‐confirmed influenza cases was in 2007 and 2009 at 23%. The proportion of influenza B was highest (51.6%) in 2010 following the A/H1N1pdm09 pandemic.^44^, ^57^, ^58^, ^59^

There were seven published articles reporting relevant data from Vietnam. Five articles reported influenza or ILI surveillance during various periods from 2001 to 2013. Two studies assessed influenza among patients hospitalized with respiratory infections. Influenza was detected in 13.9% of patients aged 15 years or older during September 2009‐August 2010, and 28.9% of all influenza viruses detected belonged to the B type.^75^

#### 3.2.16 Distribution of influenza B by age group

There were 22 articles that provided information on influenza B in different age groups (Table 4). The grouping of ages differed between studies which hinders easy comparisons.

**Table 4:** Age distribution of confirmed influenza B cases (as a percentage of all influenza B)

Country	Data source	No. of influenza B cases	Age distribution (years)						
			<5	5‐9		10‐19	20‐49	50‐64	≥65
Australia	Surveillance (Notified influenza cases)^30^	745	6.4	45 (5‐14)		46 (15‐49)		10	13
	GP surveillance^30^	38	5	24 (5‐14)		63 (15‐49)		5	3
	Surveillance (Complications alert network)^30^	50	10	8 (5‐14)		44 (15‐49)		8	30
	Clinical samples^23^	55	0	10.9		0	34.5	34.5	20.0
	Surveillance^23^	133	4.5	10.5		35.3	24.8	23.3	6.0
China	Clinical patients^135^	83	14.5	34.9 (5‐14)		25.3 (15‐24)	22.9 (25‐59)		2.4 (≥ 60)
	Clinical patients^138^	162	5.6	34.0 (5‐14)		14.2 (15‐24)	43.2 (25‐59)		3.1 (≥ 60)
	Clinical patients^133^	96	29.2	68.8 (5‐14)		0 (15‐24)	2.1 (25‐59)		0 (≥ 60)
	Clinical patients^139^	74	21.6	43.2 (5‐14)		18.9 (15‐24)	16.2 (25‐59)		0 (≥ 60)
	Clinical patients^127^	404	‐	‐ (5‐14)		29.5 (15‐24)	65.5 (25‐59)		5.0 (≥ 60)
	Clinical patients^131^	75	‐	‐ (5‐14)		50.7 (15‐24)	49.3 (25‐59)		0 (≥ 60)
	Clinical patients^41^	6688	9.7	23.5 (6‐17)		61.4 (18‐64)			5.5
Indonesia	Surveillance^43^	1487	15.0	33.8 (5‐12)		8.5 (13‐17)	38.0 (18‐49)	3.6	1.1
Laos	Surveillance^46^	142	25.4	34.8 (5‐17)			39.8 (18‐64)		0
	Surveillance^47^	92	13.0	41.3 (5‐17)			44.6 (18‐49)	1.1	0
Malaysia	Clinical patients^142^	338	73.8	16.4		8.2	1.6	‐	‐
Myanmar	Clinical patients^51^	125	43.2	32.6		13.8	2.2 (20‐59)		0 (≥ 60)
	Clinical patients^50^	6	33	50		16.7	0	0	0
PNG	Surveillance^56^	38	76	24 (>5)					
Singapore	Clinical samples^82^	92	45.3	10.5		8.2	36.0 (≥ 20)		
South Korea	Surveillance^76^	3217	48.7	18.4		5.0	7.7	6.5	13.1
Thailand	Surveillance^83^	120	25.8	43.8 (5‐17)			12.5 (18‐49)	15.6	12.5
	Surveillance^84^	150	26	34.7 (5‐17)			18.7 (18‐49)	20.7 (≥ 50)	
Vietnam	Surveillance^58^	585	22	41.5 (5‐14)			17.4 (25‐64)		1.7
	Surveillance^59^	2163	68 (0‐15)		11.8 (16‐24)		18.0 (25‐64)		1.8

N, number; PNG, Papua New Guinea; GP, general practice; ILI, influenza‐like illness; LRTI, lower respiratory tract infection. Shading indicates values covering the combined shaded age groups.

In studies reporting age‐stratified data, the proportions of influenza caused by influenza type B were higher among children aged between 1 and 10 years than in older age groups.^23^, ^30^, ^46^, ^50^, ^51^, ^56^, ^58^, ^59^, ^76^, ^77^, ^78^, ^79^, ^80^, ^81^, ^82^, ^83^, ^84^, ^85^ In 17 studies using comparable age strata, between 10.9% and 90% of all influenza B cases were detected in children aged <10 years (Table 4). Considering 18 studies with a cutoff at <20 years of age, in all but one study, more than 50% (and up to 100%) of all influenza type B cases occurred in age strata that included children and adolescents until 20 years of age. With some exceptions, in most studies (19/21), few influenza B cases (≤13.1%) were reported in adults aged 65+ years. Within the limitations of data, the age distribution of influenza B followed similar trends in each country.

One study from Victoria, Australia, estimated the rate of notified laboratory‐confirmed influenza cases (reported in 2012 to the Victorian Department of Health) to be 154/100 000 persons in those aged 0–4 years, 137/100 000 in those aged ≥65 years, and 61–90/100 000 for other age groups.^30^ The proportion of notified cases that were influenza B was highest in those aged 5‐15 years (30.3%) and 15‐29 years (20.5%), followed by those aged 30‐49 years (14.2%), 50‐64 years (10.7%), <5 years (8.7%), and ≥65 years (8.6%).

### 3.3 Circulating influenza B strains

The available data indicate that both Yamagata and Victoria influenza type B lineages have circulated in Asia‐Pacific countries during the last decade. The ratio of Yamagata to Victoria strains varied from year to year, and sometimes differed between countries in the northern and Southern Hemispheres within the same season (Table 3). Furthermore, the predominant lineage was not always the same in countries within the same region. For example, in 2012, an influenza type B Victoria strain predominated in Australia, whereas a Yamagata strain predominated in New Zealand (Table 3). In 2007, a Yamagata strain predominated in Malaysia, whereas a Victoria strain predominated in Indonesia (Table 5).

**Table 5:** Circulating and vaccine influenza type B lineages in tropical countries

Year	Vaccine lineage^15^	% type B	Circulating lineage (%)		Circulating lineage (%)		Circulating lineage (%)	
			Victoria	Yamagata	% type B	Victoria	% type B	Victoria
		Malaysia (clinical samples^49^)			Indonesia (surveillance^43^)		Laos (surveillance^46^)	
2005	Yamagata	51	99	1	‐	Predominant	‐	‐
2005‐06	Yamagata							
2006	Victoria	43	94	6	‐	Predominant	‐	‐
2006‐07	Victoria							
2007	Victoria	30	27	73	‐	Predominant	‐	‐
2007‐08	Victoria							
2008	Yamagata	18	0	100	‐	‐	66.7	‐
*2008‐09*	*Yamagata*							
2009	Yamagata	22	97	3	‐	‐	2.7	100
*2009‐10*	*Victoria*							
2010	Victoria	‐	‐	‐	‐	‐	33.7	98.4

Year		Thailand (surveillance^72^ clinical samples^73^, ^a^)			Cambodia (surveillance^32^, ^33^)		Myanmar (clinical samples^52^)	
2004	Yamagata	16	32	68	‐	‐	‐	‐
2004‐05	Victoria							
2005	Yamagata	32	41	59	‐	‐	42	85
2005‐06	Yamagata							
2006	Victoria	29	55	45	0	‐	0	‐
2006‐07	Victoria							
2007	Victoria	34	62	38	57.7	All	67	100
2007‐08	Victoria							
2008	Yamagata	40	40	60	34	None	‐	‐
2008‐09	Yamagata							
2009	Yamagata	12	100	0	12.6	All	‐	‐
2009‐10	Victoria							
2010	Victoria	35	100	0	23.1	All	‐	‐
2010‐11	Victoria							
2011	Victoria	‐	90	10	64.8	All	‐	‐
2011‐12	Victoria							
2012	Victoria	‐	50	50	‐	‐	‐	‐
2012‐13	Yamagata							
	Yamagata	‐	0	100	‐	‐	‐	‐
2013‐14	Yamagata							
2014	Yamagata	‐	0	100	‐	‐	‐	‐

a 2011‐2014 limited to 35 isolates over the 5‐year period.

In Northern Hemisphere and tropical countries, mismatches with the Northern Hemisphere vaccine occurred in 2004‐05 in Thailand; 2005‐06 in Malaysia, Indonesia, Myanmar, and Thailand; 2006‐07 in Thailand; 2007‐08 in South Korea and Indonesia; 2008‐09 in China; 2011‐12 in Taiwan; 2012‐13 in Thailand; 2012‐13 and 2013‐14 in South Korea and China; and 2013‐14 and 2014‐15 in Taiwan.

In Southern Hemisphere and tropical countries, mismatches with the Southern Hemisphere trivalent influenza vaccine occurred in 2005 in Australia, New Zealand, Malaysia, Indonesia, Thailand, and Myanmar; 2006 in Thailand; 2007 in Australia; New Zealand and Malaysia; 2008 in Australia and New Zealand; 2009 in Australia, Malaysia, Laos, Thailand, and Cambodia; 2012 in New Zealand and Thailand; and 2015 in Australia and New Zealand.

In countries that provided seasonal numerical estimates of lineage distribution, the majority of mismatched seasons in each country were significant or complete mismatches (Tables 3 and 5). In mismatched years without precise estimates of the distribution of type B lineages, the available descriptions suggest that all of the mismatches were likely to be significant or complete.

## 4 DISCUSSION

The evidence we reviewed from the published literature indicates that influenza is an important cause of morbidity in the Asia‐Pacific region and affects all age groups. Influenza type B was identified in all 15 of the countries studied, and the proportion of influenza B isolated in clinical specimens from different settings varied markedly from season to season, ranging between 0% and as high as 92%. This variability is consistent with the unpredictable seasonal influenza burden that results from co‐circulation of several types/lineages, the degree of antigenic drift, combined with the hosts’ immune status, which together ensure the continuing ability of the virus to cause illness.^9^

Significant or complete mismatches between the circulating and trivalent vaccine type B strain were observed on numerous occasions in countries with available data. Our study was not designed to quantify the possible public health implications in seasons where vaccine mismatch existed. Influenza vaccine efficacy is reduced when there is a mismatch between vaccine and circulating strains,^14^, ^86^ suggesting that a mismatch season is likely to be associated with a higher clinical disease burden.

Influenza B causes similar morbidity as influenza A.^87^ In mismatch seasons, hospitalization due to influenza type B can exceed that due to influenza A in all age groups.^71^ The available data suggest that consistent with observations in other regions,^7^, ^9^ in the Asia‐Pacific region, influenza B occurs more frequently in children aged between 1 and 10 years than in other age groups and causes more severe disease in this age group than influenza A.^88^

Evidence suggests differences in the age distribution of patients infected with the B/Yamagata or B/Victoria lineages, with the latter appearing to be more frequently identified in younger age groups.^89^, ^90^, ^91^, ^92^, ^93^ Although the lineage seems to have generally no impact on the clinical outcome of the infection, recent data from Hong Kong suggested that B/Victoria viruses may be associated with more influenza B hospitalization in children compared with B/Yamagata viruses.^94^ Both the B/Yamagata and B/Victoria lineages have been included in recommendations by WHO since September 2012 for the Southern Hemisphere vaccines for 2013 and thereafter for both hemispheres, but extensive use of quadrivalent vaccines lagged until 2015 or later, and then, it was mostly used in developed countries such as USA, Japan, Australia, and some European countries. As younger children have a globally higher probability of being infected with the influenza B viruses, this group is most likely to benefit more from a quadrivalent vaccine containing both B lineages due to the frequent mismatch and co‐circulation of both influenza B lineages.

The articles included in this review varied with respect to their design, population characteristics (eg, age range, mild vs severe cases), the illness definition selected for study (specific vs non‐specific diagnoses), the laboratory methods used to detect influenza, and the methods of case surveillance (population‐based, laboratory‐based, hospital or emergency department‐based, sentinel general practice). Therefore, the results of individual studies cannot be easily compared and are unlikely to be broadly representative. In some articles, data were not stratified per year, and only an average proportion of influenza type B over the study period could be obtained. In many studies, proportions of influenza B among any laboratory‐confirmed influenza cases were only available within a specified population. Most information was retrieved for China, Australia, South Korea, and Taiwan, but four articles or fewer were identified for Laos, Myanmar, the Philippines, and Papua New Guinea.

This review has identified important knowledge gaps within the region. In several countries, the epidemiology of influenza and influenza type B is not well described, little is known about the age groups most affected by influenza type B and the relative contribution of the two type B lineages to the disease burden. Epidemiological data for the two circulating influenza B lineages in the Asia‐Pacific region are extremely limited. Co‐circulation of Yamagata and Victoria lineages occurred in most countries where strain surveillance data were available, with considerable fluctuation from year to year. A variable, but substantial influenza B burden, as well as variable mismatch between circulating lineages and vaccine lineages was observed in all countries with available data regardless of geographical location, suggesting that a shift from trivalent to quadrivalent seasonal influenza vaccines that include both influenza B lineages would be beneficial in many seasons. Establishing or enhancing existing influenza surveillance networks in individual countries across the Asia‐Pacific region is needed to contribute to an improved understanding of the burden of influenza. Education of the medical profession and public, vaccine implementation strategies including the development of specific national recommendations in countries where they are lacking, and improved access to influenza vaccines, are needed to improve influenza vaccine uptake and reduce the influenza disease burden in Asia‐Pacific countries.

Few countries in the Asia‐Pacific region have policies, recommendations, or funding methods in place supporting influenza immunization, and those that do limit publicly funded re‐imbursement to at‐risk groups. Our review provides evidence of a substantial influenza burden in the Asia‐Pacific. The data suggest that countries in the Asia‐Pacific stand to benefit from development of immunization policy targeting influenza prevention. Additionally, quadrivalent influenza vaccines that reduce the likelihood of vaccine mismatch among influenza type B strains are likely to provide improved protection against influenza type B infection.

## CONFLICT OF INTEREST

LJ is immediate past Chairperson of the Asia Pacific Advisory Committee for Influenza Ltd. (APACI), a Charitable Trust registered in Hong Kong. He reports unrestricted research funding from F. Hoffman—La Roche and honoraria & travel assistance for participating in scientific meetings from F. Hoffman—La Roche, the GSK group of companies, Baxter, and Sanofi Pasteur, outside of the submitted work. QSH, P‐IL, and WJK report no conflict of interest. IB reports personal shares holding from an influenza vaccine manufacturer (not GSK). PB, BAM, and JC are employees of the GSK group of companies and report holding shares in the GSK group of companies as part of their employee remuneration. MS was an employee of the GSK group of companies at the time of the study.

## CONTRIBUTORSHIP

LJ, QSH, and WJK participated in the design of the review. LJ also participated in assembling and analysis of data and provided Asia‐Pacific regional knowledge for the interpretation of the published material collected. QSH, IB, P‐IL, and WJK participated in assembling and interpreting the data. MS, P‐IL, and WJK participated in the analysis and interpretation of data. PB and BAM participated in collecting, assembling, analysis, and interpretation of data. BAM also participated in the design of the review. JC participated in the design of the review, collecting the data, the supervision of the analysis, and the interpretation of data. All authors were involved in the development of this manuscript, had full access to the data, and gave final approval before submission.

## SOURCES OF SUPPORT

GlaxoSmithKline Biologicals SA was the funding source and was involved in design and conduct of the study; management, analysis, and interpretation of the data; preparation, review, and approval of the manuscript and decision to submit the manuscript for publication. Pallas was involved in the design of the study and conducted the literature review. GlaxoSmithKline Biologicals SA funded all costs associated with the development and the publishing of the present manuscript. The corresponding author had full access to the data and was responsible for submission of the publication. The Melbourne WHO Collaborating Centre for Reference and Research on Influenza is supported by the Australian Government Department of Health.
