# Supplementary material for: Literature review of the epidemiology of influenza B disease in 15 countries in the Asia‐Pacific region
Source: Influenza Other Respir Viruses. 2018 Mar 7;12(3):383–411. doi: 10.1111/irv.12522 (PMC5907823; doi:10.1111/irv.12522)
Supplement: Supplementary file 1 [file IRV-12-383-s001.docx]

**Supporting information**

**1: Search terms**

**Pubmed search strings**

**#1 Search string to identify articles on influenza:**

Human influenza[MeSH] OR flu[tw] OR influenza-like illness[tw] OR flu-like illness[tw] OR ILI[tw]

***#2. Search string to identify articles on* *countries in the Asia-Pacific region:***

Australia*[tw] OR Indonesia*[tw] OR Malaysia*[tw] OR Malay[tw] OR “New Zealand”[tw] OR

Singapore*[tw] OR Korea*[tw] OR Taiwan*[tw] OR Thailand[tw] OR Thai[tw] OR Philippines[tw] OR Filipin*[tw] OR Vietnam*[tw] OR Pacific[tw] OR Australia[ad] OR Indonesia[ad] OR Malaysia[ad] OR “New Zealand”[ad] OR Singapore[ad] OR Korea[ad] OR Taiwan[ad] OR Thailand[ad] OR Philippines[ad] OR Vietnam[ad]

***#3. Search string to identify articles on* *epidemiology:***

Incidence[MeSH] OR mortality[MeSH] OR “burden of disease”[tiab] OR “disease burden”[tiab] OR epidemiology[MeSH] OR epidemiology[subheading] OR epidemiology[tiab] OR prevalence[MeSH] OR incidence[tiab] OR prevalence[tiab] OR surveillance*[tiab] OR “sentinel surveillance”[Mesh] OR “excess morbidity”[tiab] OR morbidity[MeSH] OR morbidity[tiab] OR mortality[tiab] OR mortality[subheading] OR death*[tiab] OR “case-fatality”[tiab] OR lethal*[tiab] OR surveillance*[tiab] OR hospital*[tiab] OR “hospital

admission”[tiab] OR “hospital admissions”[tiab] OR “hospitalization rate”[tiab] OR “hospitalisation rate”[tiab] OR "GP home visits"[tiab] OR “GP visits”[tiab] OR “GP consultations”[tiab] OR "general practitioner"[tiab] OR "general practitioners"[tiab] OR “Delivery of Health Care"[mesh] OR “health care”[tiab] OR “health resources”[tiab] OR "Intensive Care"[mesh] OR “intensive care”[tiab] OR ICU[tiab] OR "Inpatients"[mesh] OR inpatient*[tiab] OR visit*[tiab] OR complication*[tiab] OR ambulator*[tiab] OR GP[tiab] OR “disease outcome”[tiab] OR “emergency room”[tiab] OR “emergency department”[tiab] OR

“primary care clinic”[tiab] OR “ER visits”[tiab] OR “ED visits”[tiab] OR pneumonia[tw] OR respiratory disease*[tw] OR respiratory complication*[tw] OR respiratory disorder*[tw] OR respiratory tract infection*[tw]

The search strings were combined as followed: #1 AND #2 AND #3, and the search was carried out on 23-07-2013.

**Western Pacific Region Index Medicus search strings**

A WPRIM search was performed by adapting PubMed search strings #1 and #2. No search string on epidemiology was included for the WPRIM search, as the number of hits retrieved by combining search string #1 and #2 was already limited.

**#1 Search string to identify articles on influenza:**

All:human influenza of All:flu or All:influenza-like-illness or All:ILI or All:influenza

**#2. Search string to identify articles on countries in the Asia-Pacific region:**

All:Australia% or All:Indonesia% or All:Malaysia% or All:Malay or All:”New Zealand” or All:Singapore% or All:Korea% or All:Taiwan% or All:Thailand or All:Thai or All:Philippines or All:Filipin% or All:Vietnam% or All:Pacific

The search strings were combined as followed: #1 AND #2, and the search was last carried out on 04-04-2016.
